# The role of interferon regulatory factors in atherosclerosis

**DOI:** 10.3389/fcvm.2025.1606034

**Published:** 2025-09-04

**Authors:** Yingsong Wang, Tianxiang Fang, Xiaoya Zheng, Ning Huangfu

**Affiliations:** ^1^ Department of Cardiology, The First Affiliated Hospital of Ningbo University, Ningbo, China; ^2^ Health Science Center, Ningbo University, Ningbo, China; ^3^ Key Laboratory of Precision Medicine for Atherosclerotic Diseases of Zhejiang Province, Department of Cardiology, Ningbo, China; ^4^ Clinical Medicine Research Centre for Cardiovascular Disease of Ningbo, Ningbo, China

**Keywords:** interferon regulatory factors, atherosclerosis, inflammation, lipid metabolism, cell death

## Abstract

Cardiovascular diseases (CVDs) remain a leading cause of global morbidity and mortality, largely driven by the progression of atherosclerotic plaques. In atherosclerosis (AS), transcription factors and epigenetic mechanisms play pivotal roles in regulating gene expression. Interferon regulatory factors (IRFs), a family of transcription factors initially identified for their role in coordinating interferon (IFN) responses, are now recognized as critical modulators of innate and adaptive immunity. Emerging evidences highlights their involvement in inflammation, lipid metabolism, cell differentiation, cell proliferation, and programmed cell death during AS pathogenesis. This review synthesizes current knowledge on the roles and regulatory mechanisms of IRFs in AS, offering novel insights and potential therapeutic targets for AS management.

## 1 Introduction

Cardiovascular diseases (CVDs) remain a leading cause of global morbidity and mortality, which projected to account for 23.6 million annual deaths by 2030 (1). Atherosclerosis (AS) serves as the primary pathological basis for most CVD-related fatalities. It is a chronic inflammatory disorder characterized by endothelial dysfunction, lipid accumulation, and immune cell infiltration. During early atherogenesis, specific plasma lipoproteins, including low-density lipoprotein (LDL), lipoprotein(a), and triglyceride-rich lipoprotein remnants may undergo modifications within the arterial intima. This process initiates an inflammatory cascade that drives monocyte recruitment and their subsequent differentiation into macrophages. Within this microenvironment, macrophages phagocytose modified lipoprotein particles, forming foam cells that progressively accumulate lipids, cholesterol esters, and cellular debris. Concurrently, vascular smooth muscle cells (VSMCs) migrate to the intima, proliferate, and contribute to plaque calcification (2). These events are tightly regulated by transcriptional and epigenetic mechanisms, with transcription factors such as Interferon regulatory factors (IRFs) emerging as key players in AS progression (3).

The IRFs are a family of transcription factors that are highly conserved across species. Originally identified as regulators of IFN signaling, IRFs (IRF1–IRF9) have expanded roles in immune responses, lipid metabolism, and cellular stress. They are extensively involved in diverse biological processes, including cytokine signaling, pathogen response, cell cycle regulation, cell differentiation, apoptosis, and hematopoietic development (4–9). Emerging evidence highlights their role in AS-related processes, including endothelial activation, macrophage polarization, and VSMC dysfunction (3, 10–12). However, the specific roles and mechanisms haven't yet to be fully elucidated. This review comprehensively examines the contributions of IRF family members to AS pathogenesis, emphasizing their potential as therapeutic targets.

## 2 Interferon regulatory factors: structure and function

**Figure 1:**
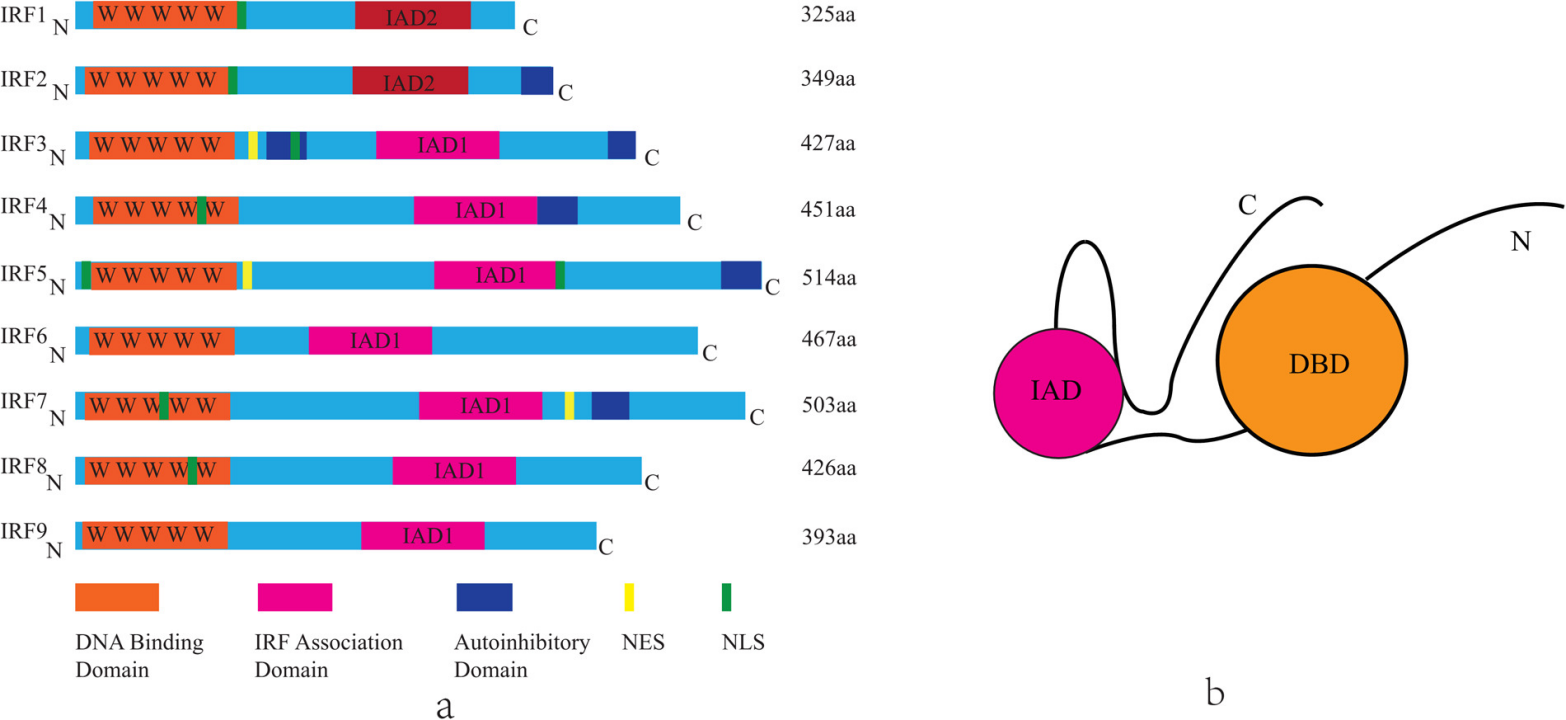
The structure of mammalian IRF family members. **(a)** Each IRF contains a highly conserved N-terminal DNA-binding domain composed of five tryptophan repeat sequences(DBD, orange). In the C-terminal region, IRF1 and IRF2 share the IRF association domain 2 (IAD2, pink), while the other IRFs share the IRF association domain 1 (IAD1, pink). Other domains include the nuclear localization signal (NLS, green), the nuclear export signal (NES, yellow), the autoinhibitory domain (blue). The length of each IRF is represented by the number of amino acids (aa), found in Uniprot. IRF, interferon regulatory factor; C, carboxy terminus; N, amino terminus. **(b)** Schematic illustration of the structure of the IRFs.

The mammalian IRF family comprises nine members (IRF1- IRF9), each containing a conserved N-terminal DNA-binding domain (DBD) and a C-terminal IRF-association domain (IAD) (Figure 1) (13, 14). While the DBD facilitates recognition of IFN-stimulated response elements (ISREs), the IAD mediates interactions with cofactors, enabling diverse regulatory functions (Table 1) (15, 16). Key signaling pathways, such as Toll-like receptor (TLR) and cytosolic DNA-sensing cascades (e.g., cGAS-STING), converge on IRFs to modulate inflammatory and metabolic responses (17).

**Table 1:** Classification of IRF family.

IRFs member	Main functions	References
IRF1	Orchestrates innate immune responses and antiviral defense; modulates immune cell differentiation, apoptosis, and anti-proliferative activity across hematopoietic lineages.	(14, 22–24)
IRF2	Regulates immune cell maturation and transcriptional activation/repression in hematopoietic development.	(25)
IRF3	Key mediator of innate antiviral signaling; initiates type I interferon production via pathogen-sensing pathways.	(26, 27)
IRF4	Modulates oncogenic processes and lipid homeostasis in adipocytes; fine-tunes adaptive immune responses.	(28–32)
IRF5	Central driver of autoimmune pathogenesis; regulates pro-inflammatory cytokine production and macrophage polarization.	(34–40)
IRF6	Governs epithelial differentiation and morphogenesis; critical for epidermal and craniofacial development.	(42, 43)
IRF7	Master regulator of type I interferon amplification; essential for antiviral innate immunity and dendritic cell activation.	(44, 45)
IRF8	Controls myeloid/lymphoid cell differentiation; regulates antigen presentation and inflammasome activation.	(46–48)
IRF9	Mediates interferon signaling; emerging roles in metabolic regulation (hepatic steatosis, insulin resistance).	(49, 50)

IRF activation progresses through five core phases: (1) signal perception, (2) post-translational modifications (PTMs), (3) dimerization, (4) nuclear translocation, and (5) transcriptional regulation. Crucially, PTMs serve as the initiating molecular switch, with phosphorylation, acetylation, ubiquitination, and SUMOylation being principal modifications that dynamically regulate IRF functional states (17). Subsequently, homo- or hetero-dimerization constitutes an essential activation prerequisite, enabling conformational changes required for nuclear trafficking (18). Latent cytoplasmic IRFs undergo stimulus-dependent nuclear translocation upon PRR activation. These receptors—including TLRs, NLRs, RLRs, and DNA sensors—detect PAMPs/DAMPs to trigger IRF-mediated IFN/pro-inflammatory responses, demonstrating dual roles in AS pathogenesis (protective vs. pathogenic) (19–21). The TLR pathway bifurcates into TRIF- and MyD88-dependent arms, with IRF members exhibiting selective or combinatorial pathway activation to drive context-specific transcriptional programs (17).

### 2.1 Key IRF family members

IRF1: a pleiotropic transcription factor predominantly localized within the nuclear compartment. Its expression undergoes significant upregulation following viral infection or immune stimulation, mediated through critical signaling pathways IFN(Interferon), NF-*κ*B(Nuclear Factor kappa B), TBK-1(TANK-Binding Kinase 1), and IKK-ɛ(Inhibitor of Nuclear Factor Kappa-B Kinase Subunit ɛ). This transcription factor plays a pivotal role in orchestrating the development, differentiation, and functional regulation of key immune cell populations, particularly B lymphocytes, T helper 1 (Th1) cells, and dendritic cells (DCs). Additionally, IRF1 exerts critical cytostatic and pro-apoptotic effects across diverse mammalian cell types. Mechanistically, these activities involve modulation of oxidative stress responses and participation in regulated cell death pathways, including ferroptosis (14, 22–24).

IRF2: a competing factor engages in cis-regulatory element occupation, directly antagonizing IRF1-mediated transcriptional activity by binding to shared DNA recognition motifs. This molecular interference suppresses the expression of IRF1-dependent pro-inflammatory genes, while simultaneously fine-tuning transcriptional programs critical for immune cell ontogeny and functional maturation including lineage commitment, antigen presentation capacity, and effector molecule production. Such competitive regulation establishes a dynamic equilibrium between inflammatory activation and homeostatic restraint, ensuring balanced immune responses while preventing pathological hyperinflammation (25).

IRF3: primarily resides in the cytoplasm in an inactive state, serving as an essential component of the innate immune defense system. This transcription factor demonstrates remarkable sensitivity in detecting viral components and plays a pivotal role in initiating antiviral responses to prevent pathogenic infections. Furthermore, recent studies have revealed IRF3's significant involvement in cardiovascular pathophysiology, where it functions as a negative regulator of pathological cardiac remodeling. Mechanistically, IRF3 binds to Extracellular Signal-Regulated Kinase(ERK) 2 through protein-protein interaction, leading to subsequent suppression of ERK 1/2 signaling activity (26, 27). This regulatory mechanism not only highlights IRF3's dual functionality in both immune regulation and cardiac homeostasis but also provides potential therapeutic targets for CVDs.

IRF4: initially characterized as a lymphocyte-specific nuclear factor and discovered to exhibit conserved cardiac expression in both human and animal models (28). Distinct from other IRF family members, IRF4 demonstrates unique activation mechanisms, primarily responding to mitogenic stimuli such as antigen receptor signaling, lipopolysaccharide (LPS) stimulation, and CD40 receptor engagement rather than canonical interferon-mediated pathways (29, 30). Beyond its established role as an oncogenic transcription factor that modulates upstream signaling cascades and protein-DNA interactions, IRF4 paradoxically serves dual functions in metabolic regulation. It acts as a master transcriptional regulator of lipid homeostasis in adipocytes and functions as an anti-inflammatory mediator in diet-induced obesity, positioning it at the intersection of metabolic inflammation and energy balance (31, 32).

IRF5: predominantly expressed in immune cells such as monocytes, macrophages, B lymphocytes, and DCs. Its dysregulation is strongly implicated in the pathogenesis of autoimmune disorders including systemic lupus erythematosus (SLE), inflammatory bowel disease (IBD), and rheumatoid arthritis (RA) (33). his transcription factor plays a pivotal role in inflammatory responses through its synergistic interaction with the NF-*κ*B p65 subunit RelA, co-activating inflammatory gene networks and driving the production of key pro-inflammatory cytokines such as interleukin-6 (IL-6), IL-12, and tumor necrosis factor-alpha (TNF-α) (34–36). Emerging genetic evidence further underscores the clinical significance of IRF5 polymorphisms, which have been associated with both susceptibility to and protection against AS. Notably, the high degree of genetic variation in IRF5 correlates with pathological changes in carotid intima-media thickness (cIMT) and demonstrates a strong association with coronary artery disease (CAD) development in SLE patients (37–40).

IRF6: critically regulates epidermal development and differentiation (41). Emerging evidence further suggests its potential involvement in the pathogenesis and metabolic reprogramming of pancreatic ductal adenocarcinoma and gliomas (42, 43).

IRF7: a key regulatory component in type I/III IFN-mediated signaling cascades. It shares significant structural homology with IRF3 while exhibiting distinct functional specialization. Notably, it serves multifaceted roles in antiviral defense mechanisms and innate immune responses (44). Emerging evidence implicates IRF7 in modulating obesity-associated adipose tissue inflammation (45).

IRF8: originally characterized as being selectively expressed in immune system lineages (lymphoid and myeloid cells). It serves as a master regulator of immune cell development and maturation (14). Additionally, IRF8 acts as a potent inducer of macrophage differentiation from bone marrow progenitor cells. Genome-wide association studies have revealed significant correlations between IRF8 genetic variants and CAD manifestations, particularly through three key phenotypic markers: carotid plaque formation, augmented carotid intima-media thickness, and systemic leukocytosis. These clinical observations collectively establish its potential role as a biomarker for subclinical AS risk (46–48).

IRF9: a critical downstream effector of type I IFN signaling through its integration into the STAT1-STAT2 heterotrimeric complex, collectively forming the interferon-stimulated gene factor 3 (ISGF3) transcriptional machinery. While its canonical role in IFN response pathways is well-established, its specific regulatory mechanisms governing immune cell ontogeny remain underexplored (49). Notably, IRF9 interacts with peroxisome proliferator activating receptor alpha (PPAR*α*) and forms a metabolic-regulator*y* axis that co-activates PPAR*α*-responsive genes. This interaction manifests therapeutic potential by ameliorating pathological processes including inflammatory responses, hepatic lipid accumulation, and insulin resistance (50).

## 3 IRFs and as

Current observations highlight a significant role for IRFs in murine models of atherosclerosis AS. In apolipoprotein E-deficient (ApoE^−/−^) mice, IRF1 promotes a proinflammatory M1 macrophage phenotype, exacerbates atherosclerotic burden (51), facilitates foam cell formation and increases plaque instability (3). Conversely, ablation of IRF3 expression enhances plaque stability, characterized by thicker fibrous caps, increased collagen deposition and smooth muscle cell (SMC) content, alongside reduced macrophage infiltration (10, 52). Similar to IRF1, IRF5 can also promotes macrophage polarization towards a pro-atherogenic state and foam cell formation (53–55). Notably, IRF7 plays a non-redundant role in AS pathogenesis specifically in diabetic mouse models (56). Furthermore, in chronic myelogenous leukemia-prone mice, IRF8 is implicated in regulating phagocytic function of macrophages (57). Whether these specific roles of IRFs translate identically to humans, beyond *in vitro* and animal studies, remains to be elucidated. The mechanisms by which IRFs contribute to AS are summarized below.

### 3.1 Inflammatory regulation

The inflammatory cascade permeates all stages of AS, with endothelial cells (ECs) and immune cells coordinating vascular inflammation through IRF-mediated mechanisms. Key IRF members demonstrate cell-type specific pro-atherogenic effects: IRF1/IRF3 modulate cytokine production in ECs and T cells, IRF5/IRF8 drive macrophage polarization, while IRF7 exhibits context-dependent anti-inflammatory properties (Table 2).

**Table 2:** The role of IRFs in inflammtory response.

Irfs	Influenced genes	Involved signal pathway	Effects	References
IRF1	VCAM-1	Activates: PERK-eIF2*α*-CHOP pathway, PECAM-1-P38-XBPs pathway	Promotes EC inflammation	(61, 62, 65)
		Inhibits: IL-33-ERK1/2 pathway; Ataxin-10		(66, 67)
	CD40	-	Induces adhesion molecules and chemokines expression in ECs	(68)
	RIG-I	MAVS/TAK-1/JNK&ERK1/2 signaling pathway	Stimulates IL-8 secretion in macrophages	(69)
	TNF-α, IL-1β, CCL-2	TLR2/TLR4/SUB1 pathways	Drives macrophage polarization to M1 phenotype	(51)
	IFN-*γ*, TNF-α, IL-12p70	-	Enhances Th1-mediated pro-inflammatory cytokine production	(70)
	IL-6, IL-12p70, TNF-α	MAPK pathway (JNK/p38/ERK1/2 phosphorylation)	Upregulates inflammatory cytokines and enhances DC maturation	(71)
IRF3		Noncanonical STING-PERK pathway (complex with NF-*κ*B & BRD4)	Mediates EC inflammatory activation	(72)
	IL-6	cGAS-STING DNA-sensing pathway	Promotes VSMC inflammation.	(73)
	IL-6, PPARγ	MyD88-independent TLR4 signaling	Contributes to VSMC inflammatory responses	(75)
	CCL5, Cxcl10	IKK-related kinase/TBK1 pathway	Upregulates RANTES and IP-10 expression	(74)
IRF5	IL-10	TLR7/TLR9 pathway	Attenuates macrophage inflammation in lupus-associated atherosclerosis	(80)
	CD11c, CCL2, CCL4	-	Activates macrophage inflammatory responses	(78, 79)
	TNF-α, IL-6	TRAF6-IKK-IRF5 axis	Drives pro-inflammatory gene expression in M1 macrophages	(54, 55)
IRF7	iNOS, Arg1, IL-10	-	Modulates macrophage inflammatory processes	(56)
IRF8	Lymphoid CD8α+ cDCs	Aortic CD11b–CD103 + cDCs	Regulates dendritic cell subpopulation development	(82)
	Arg1	-	Suppresses macrophage inflammatory activity	(81)

#### 3.1.1 IRF 1

Inflammation of damaged ECs leads to endothelial dysfunction, a critical initiating event in atherosclerotic plaque development and progression (58). IRF1 orchestrates endothelial dysfunction through multiple mechanosensitive pathways, though its role in the atherosclerotic microenvironment remains controversial. Previous studies have reported conflicting IRF1-dependent regulation of TNF-induced vascular cell adhesion molecule-1 (VCAM-1) expression (59, 60). Emerging evidence clarifies that IRF1 mediates triglyceride-rich lipoprotein (TGRL)-induced VCAM-1 via the PERK/eIF2*α*/CHOP signaling axis, with polyunsaturated fatty acids counteracting this pro-inflammatory pathway in human aortic endothelial cells(HAECs) (61, 62). Shear stress (SS), the frictional force exerted by blood flow on ECs, modulates IRF1 activity through distinct mechanisms (63). Fluid SS regulates IRF1 nuclear translocation via platelet EC adhesion molecule 1 (PECAM-1)-mediated mechanotransduction, involving p38/x-box binding protein 1 (XBP1s) interactions and Phosphoinositide 3-kinase (PI3 K)/MAPK phosphatase 1 (MKP-1) modulation (64, 65). Conversely, interleukin 33 (IL-33) inhibits VCAM-1 expression through IRF1- ERK 1/2 pathway cross-talk in HAECs (66). Additionally, Ataxin-10 binds cytoplasmic IRF1 to suppress its nuclear translocation and downstream cytokine/chemokine transcription (67). Moreover, IRF1 drives CD40(TNFR5) expression in ECs, promoting adhesion molecules and chemokines essential for leukocyte recruitment (68). By constructing a mouse atherosclerotic model, Wang et al. found in human and mouse primary cells that the IRF1/RIG-I axis mediates 25-hydroxycholesterol-induced IL-8 production in AS, activating NF-*κ*B, AP-1, and NF-IL-6 through MAVS/TAK-1/JNK/ERK1/2 signaling (69). In ApoE^−/−^ mice, IRF1 promotes M1 polarization via TLR2/4-casein kinase 2 (CK2)- RNA polymerase II transcriptional coactivator p15 (SUB1/Sub1, PC4) signaling, enhancing TNF-α/IL-1β production (51). This pro-inflammatory shift exacerbates plaque vulnerability. IRF1-driven IFN-*γ*/TNF-α production contributes to plaque rupture and acute coronary syndromes (ACS) (70). In rat VSMCs, IRF1 amplifies DC inflammatory responses through MAPK pathway activation (JNK/p38/ERK1/2 phosphorylation), accelerating plaque destabilization (71).

#### 3.1.2 IRF 3

IRF3 demonstrates paradoxical regulatory effects in vascular inflammation through distinct molecular mechanisms (72). Knockdown of EC-specific IRF3 significantly attenuates plaque formation in Western diet-fed ApoE^−/−^ mice, while bone marrow-derived macrophage IRF3 shows no such effect. Mechanistic studies demonstrate that IRF3 specifically binds to the ISRE within the ICAM-1 promoter(P1 site), predominantly suppressing ICAM-1 transcription and moderately inhibiting VCAM-1 expression (10, 52). Paradoxically, while suppressing ICAM-1 transcription through ISRE binding, IRF3 simultaneously promotes inflammatory signaling through four distinct axes.

##### 3.1.2.1 Axis 1: cGAS-STING-PERK activation via oxidized mtDNA

Oxidized mitochondrial DNA triggers cyclic GMP-AMP (cGAMP) synthase (cGAS)-stimulator of IFN genes (STING) signaling, leading to the formation of a transcriptional complex comprising IRF3, NF-*κ*B, and bromodomain protein 4 (BRD4). This complex drives pro-inflammatory genes expression including ICAM-1, through PERK pathway activation in both human and mouse ECs (72).

##### 3.1.2.2 Axis 2: cGAS-STING-IRF3-Il6 activation via DNA double-strand breaks

In ApoE^−/−^ mice, DNA damage-induced double-strand breaks (DSBs) activate this axis to amplify pro-inflammatory responses in AS, highlighting its potential as a therapeutic target (73).

##### 3.1.2.3 Axis 3: TBK1/IRF3/CCL5-CXCL10 activation in HCMV-infected VSMCs

Human cytomegalovirus (HCMV) infection in human VSMCs activates a novel I*κ*B kinase (IKK)-related pathway involving TBK1, IRF3, and chemokines CCL5/RANTES and CXCL10/IP-10. This axis contributes to atherosclerotic lesion progression by modulating inflammatory chemokine production (74).

##### 3.1.2.4 Axis 4: TLR4-TRIF-Ip10 pathway modulation

IRF3 mediates C-reactive protein (CRP)-induced NF-*κ*B activation in rat VSMCs via the MyD88-independent TLR4 pathway. This mechanism suppresses peroxisome proliferator-activated receptor *γ* (PPAR*γ*) expression, a key anti-inflammatory regulator while enhancing IL-6 production. Notably, the PPAR*γ* agonist rosiglitazone reverses PPAR*γ* downregulation and demonstrates novel anti-inflammatory effects through modulation of the TLR4/TRIF/IRF3/IP-10 signaling axis (75, 76).

Recent work by Zeyu Xing et al. reveals that karyopherin subunit alpha 2 (KPNA2) facilitates nuclear translocation of IRF3 and NF-*κ*B p65,.a process regulated by the E3 ubiquitin ligase F-box and WD repeat domain containing 7 (FBXW7) (77). This discovery expands our understanding of post-translational regulation in IRF3-mediated inflammatory responses.

#### 3.1.3 IRF5/7/8

IRF5 is well-established as a driver of pro-inflammatory M1 macrophage polarization. Emerging evidence highlights its paradoxical role in inflammatory regulation. Julia Leipner et al. demonstrated that IRF5 deficiency reduces M1 marker expression *in vitro* and enhances atherosclerotic plaque stability, suggesting its critical role in sustaining vascular inflammation (53). Mechanistically, in ApoE^−/−^ mice, IRF5 promotes M1 polarization via TRAF6-IKK and miR-22-dependent pathways, positioning it as a promising therapeutic target for AS management (54, 55). IRF5 further induces the differentiation of macrophages into the pro-inflammatory CD11c + subset by directly targeting the CD11c gene, thereby amplifying chemokine release (CCL2 and CCL4) to fuel inflammatory cascades (78, 79). Notably, IRF5 exhibits disease-specific functionality. TLR7/9-activated IRF5 elevates IL-10 in systemic lupus erythematosus (SLE) models, contrasting with its pro-inflammatory role in TLR2/4-mediated pathways (80).

In diabetic contexts of mice, IRF7 disrupts macrophage homeostasis by suppressing the anti-inflammatory M2 marker arginase-1 (Arg1) and IL-10 via RAGE signaling, while enhancing pro-inflammatory TNF-α and CCL2 production (56). Conversely, IRF8 cooperates with the Ets family transcription factor PU.1, a downstream effector of liver X receptors (LXRs), to regulate Arg1 expression, highlighting its role in balancing macrophage functional states (81).

Conventional dendritic cells (cDCs) in atherosclerotic plaques, characterized by the CD11b^−^ CD103^+^ IRF8hi phenotype, originate from DNGR1-expressing precursors. Conditional deletion of IRF8 in CD11c^+^ cells ablates lymphoid-like CD8*α*^+^ cDCs and CD11b-CD103 + cDCs, significantly attenuating AS progression despite hypercholesterolemia. This effect correlates with suppressed T/B cell activation and differentiation under high-fat diet (HFD) conditions, underscoring IRF8's non-redundant role in bridging innate and adaptive immune responses in AS pathogenesis (82).

### 3.2 Dysregulation of lipid homeostasis

**Figure 2:**
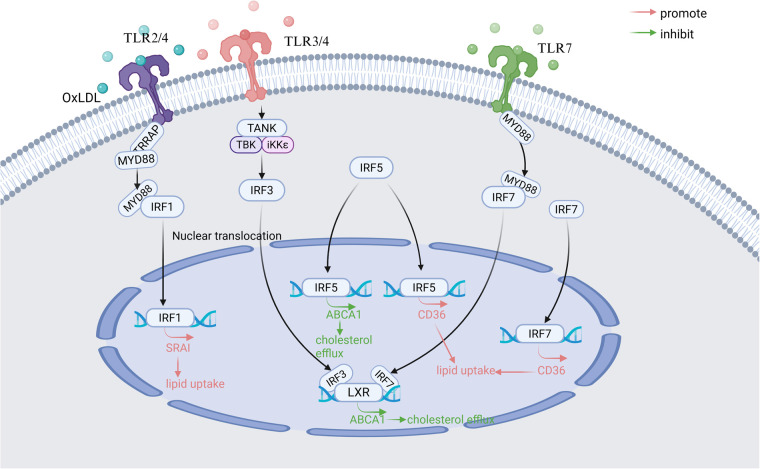
Main signaling pathways of IRFs involved in regulating macrophage lipid metabolism in atherosclerosis. TLR, Toll-like receptor; MyD88, Myeloid differentiation primary response 88; TANK, TRAF family member-associated NF-kappa-B activator; TBK-1, TANK-binding kinase 1; LXR, Liver X Receptor; IKK-*ε*, Inhibitor of nuclear factor kappa-B kinase subunit epsilon; SR-AI, scavenger receptor AI; ABCA1, ATP-binding cassette transporter A1; CD36, Cluster of Differentiation 36. Created in BioRender. Wang, Y. (2025) https://BioRender.com/zmlx50r.

Under physiological conditions, macrophages maintain lipid homeostasis through balanced uptake, efflux, and degradation. Foam cell formation, a hallmark of early AS, is tightly linked to dysregulated lipid metabolism in macrophages (83). Emerging evidence implicates IRF1, IRF3, IRF5, and IRF7 as critical regulators of lipid handling in macrophages, with distinct roles in modulating scavenger receptors, cholesterol transporters, and nuclear receptor signaling (Figure 2).

#### 3.2.1 IRF1

Elevated IRF1 expression correlates with atherosclerotic lesion progression and oxidized LDL (oxLDL)-induced foam cell formation (84). Scavenger receptor AI (SR-AI), which is a direct downstream gene target of IRF1, is primarily responsible for the recognition and uptake of oxLDL (85). Mechanistically, in a mouse atherosclerotic model, IRF1 amplifies lipid uptake in macrophages by upregulating scavenger receptor AI (SR-AI) through the TLR2/4-MyD88 pathway, while IRF1 silencing reverses this opposite effect (3). OxLDL receptor-1 (LOX-1), a transmembrane glycoprotein, is also famous for binding to and internalizing oxLDL (71). In DCs in acute coronary syndrome(ACS) patients, IRF1 overexpression enhances lectin-like LOX-1 expression, promoting oxLDL binding and internalization (71).

#### 3.2.2 IRF3

IRF3 exerts dual regulatory effects on lipid metabolism. ATP-binding cassette transporter A1 (ABCA1) is a key transporter protein for cholesterol efflux in macrophages (86). IRF3 suppresses the transcriptional activity of Liver × Receptor (LXR) at the ABCA1 promoter through TLR3/4 signaling in aortic tissue *in vivo*. This process could reduce cholesterol efflux and favor lipid accumulation (87). Concurrently, IRF3 synergizes with Chlamydia pneumoniae-activated TLR2/4 signaling via MyD88 and TRIF-dependent pathways to drive foam cell formation. Intriguingly, crosstalk between IRF3 and LXR pathways suggests therapeutic potential for LXR agonists in mitigating AS progression (88).

#### 3.2.3 IRF5

Cluster of Differentiation 36 (CD36) is a class B scavenger receptor primarily responsible for lipid uptake (89). Demonstrated *in vivo* and *in vitro* models, IRF5 promotes foam cells formation by skewing the ABCA1/CD36 ratio toward lipid uptake. This means IRF5 can reduce the expression of ABCA1 and increase the expression of CD36 in macrophages (53). Paradoxically, in SLE, IRF5 improves systemic lipid profiles by lowering very low-density lipoprotein (VLDL) and elevating high-density lipoprotein (HDL) levels, highlighting its context-dependent functionality (80).

#### 3.2.4 IRF7

IRF7 disrupts cholesterol homeostasis through the TLR7-MyD88-ABCA1 axis, suppressing cholesterol efflux in murine macrophages (90). In diabetic mouse bone marrow-derived macrophages (BMDMs), IRF7 downregulation increases cholesterol transporter expression while reducing CD36 levels, implicating that IRF7 is a negative regulator of reverse cholesterol transport (56).

### 3.3 Regulation of programmed cell death in atherosclerosis

Programmed cell death (e.g., pyroptosis, apoptosis) and efferocytosis, the phagocytic clearance of apoptotic cells, are critical determinants of atherosclerotic plaque stability. Emerging evidence highlights that exacerbated programmed cell death coupled with impaired efferocytosis drives atherosclerotic progression. Key IRF family members regulate these processes through distinct mechanisms (Table 3).

**Table 3:** The role of IRFs in programmed cell death, efferocytosis and vascular smooth muscle cells.

IRFs	Target genes	Functions	References
Programmed cell death and Efferocytosis			
IRF1	GSDMD	Induces EC apoptosis.	(11)
	NLRP3-ASC, caspase-1 p10, IL-1β p17, IL-18	Mediates macrophage pyroptosis activation	(92)
	METTL3	Enhances pyroptotic pathway activation in macrophages	(93, 94)
IRF5	Itgb3, Mfge8	Impairs macrophage efferocytic capacity	(78, 79)
IRF8	CD36	Suppresses macrophage efferocytosis	(57)
Vascular smooth muscle cells: differentiation, proliferation and calcification			
IRF1	CDK	Induces cell cycle arrest in VSMCs at G1 phase	(99)
	NOS2	Mediates VSMC cycle arrest through nitric oxide signaling	(99)
	CCL19	Facilitates VSMC pathological processes:As a potential stimulus for SMC-to-macrophage transdifferentiation.	(101)
IRF8	Unknown	Potential mediator of SMC-to-macrophage transdifferentiation	(12)

#### 3.3.1 IRF1

Pyroptosis, a caspase-dependent inflammatory cell death mechanism, is critically regulated by IRF1 (91). In HAECs, IRF1 binds promoter regions of Gasdermin D (GSDMD) and CASP1, enhancing their expression to drive NLRP3 inflammasome-mediated pyroptosis. This progress is initiated by RelB/p52-mediated NF-*κ*B activation (11). Similarly, in macrophages, IRF1 modulates Cysteine-aspartic proteases 1 (caspase-1) activation, GSDMD-N cleavage, and IL-1β/IL-18 maturation via ROS-dependent NLRP3-ASC inflammasome. IRF1 overexpression amplifies pyroptotic signaling, whereas its inhibition attenuates inflammatory cell death (92).

Notably, IRF1 further promotes macrophage from patients with CAD pyroptosis by regulating Methyltransferase-like 3 (METTL3)-mediated m6A modification of circular RNA circ_0029589. This interaction suppresses circ_0029589 expression, which normally inhibits caspase-1 p10, GSDMD-N, IL-1β p17, and IL-18 production (93, 94). IRF1 also facilitates NLRP3-ASC recruitment to enhance caspase-1 activation, establishing a feedforward loop of inflammation (93, 94).

#### 3.3.2 IRF5

In human carotid artery and mouse models, IRF5 impairs macrophage efferocytosis by downregulating integrin-*β*3 (Itgb3) and milk fat globule epidermal growth factor 8 (Mfge8), the key mediators of apoptotic cells, in pro-inflammatory CD11c + macrophages. This defect enlarges the necrotic core and increases plaque rupture susceptibility, underscoring IRF5's role in destabilizing advanced lesions (78, 79).

#### 3.3.3 IRF8

IRF8-deficient macrophages from chronic myelogenous leukemia-prone mice exhibit reduced CD36 expression, impairing efferocytosis of apoptotic polymorphonuclear neutrophilic leukocytes (PMNs) (57). This finding positions IRF8 as a regulator of phagocytic function in inflammatory contexts.

### 3.4 VSMC plasticity and phenotypic switching in atherosclerosis

VSMCs undergo dynamic phenotypic changes during AS, migrating from the medial layer to the intima, proliferating, and depositing extracellular matrix to shape plaque architecture (95). Emerging evidence highlights the regulatory roles of IRFs in VSMC plasticity, which governs their transition into distinct functional subtypes with divergent roles in AS progression (96) (Table 3).

#### 3.4.1 IRF1

IRF1 exerts antiproliferative and pro-apoptotic effects across vascular cell types (3). In murine neointimal hyperplasia models, IRF1 suppresses coronary artery smooth muscle cell (CASMC) proliferation and migration while inhibiting neointima formation. Mechanistically, IRF1 induces G1-phase cell cycle arrest via two complementary pathways: (1)P21-Dependent CDK Inhibition: IRF1 upregulates the cyclin-dependent kinase (CDK) inhibitor p21, directly or indirectly blocking CDK activity (97, 98); (2)Nitric Oxide (NO)-Mediated Arrest: IRF1 enhances NO production, a known inducer of cell cycle arrest and endothelial function modulator (99).The p21 pathway also inhibits CASMC migration, potentially through interactions with CDK inhibitory proteins (CKIs) of the Cip/Kip family (100). These dual mechanisms provide novel insights into IRF1's role in mitigating VSMC-driven AS pathophysiology.Paradoxically, IRF1 promotes AS progression by upregulating CCL19, a chemokine elevated in AS patient serum. CCL19 enhances VSMC proliferation, migration, inflammatory factor secretion (IL-1α/β, TNF-α, IL-6), and extracellular matrix deposition (collagen III, osteopontin), positioning it as a potential diagnostic biomarker and therapeutic target (101).

#### 3.4.2 IRF8

Single-cell transcriptomic analyses reveal five VSMC subtypes in AS: contractile, fibroblast-like, osteogenic, synthetic, and macrophage-like. Notably, Xue Gong et al. identified IRF8 as a master regulator SMC-to-macrophage transdifferentiation via NF-*κ*B signaling activation in human carotid plaque laden with atherosclerotic core (12). The macrophage-like SMC subtype exhibits a hybrid phenotype co-expressing inflammatory mediators(CCL2, CXCL1-3), matrix-remodeling enzymes(MMP3, MMP9, MMP19) and osteogenic markers(LGALS3, KLF4) (102–105). This transdifferentiation process amplifies plaque instability by promoting matrix degradation, inflammation, and calcification, underscoring IRF8's pathogenic role in advanced AS.

## 4 Conclusions

IRFs are multifaceted regulators of AS, influencing inflammation, lipid handling, cell death, and VSMC plasticity. While IRF1, IRF3, IRF5, and IRF8 predominantly exacerbate AS, context-dependent roles (e.g., IRF5 in lupus-associated AS) highlight their therapeutic complexity. Future studies must clarify unresolved questions, including functional redundancies among IRFs, tissue-specific effects, and translational potential. Targeting IRF signaling pathways may offer novel strategies for stabilizing atherosclerotic plaques and reducing CVD burden.

## References

[1] Tsao CW Aday AW Almarzooq ZI Anderson CAM Arora P Avery CL Heart disease and stroke statistics-2023 update: a report from the American Heart Association. Circulation. (2023) 147(8):e93–e621. 10.1161/CIR.0000000000001123 36695182 PMC12135016

[2] Libby P Buring JE Badimon L Hansson GK Deanfield J Bittencourt MS Atherosclerosis. Nat Rev Dis Primers. (2019) 5(1):56. 10.1038/s41572-019-0106-z 31420554

[3] Du M Wang X Mao X Yang L Huang K Zhang F Absence of interferon regulatory factor 1 protects against atherosclerosis in apolipoprotein E-deficient mice. Theranostics. (2019) 9(16):4688–703. 10.7150/thno.36862 31367250 PMC6643443

[4] Stoy N. Involvement of interleukin-1 receptor-associated kinase 4 and interferon regulatory factor 5 in the immunopathogenesis of sars-cov-2 infection: implications for the treatment of COVID-19. Front Immunol. (2021) 12(1664-3224):638446. 10.3389/fimmu.2021.638446 33936053 PMC8085890

[5] Luo WW Tong Z Cao P Wang FB Liu Y Zheng ZQ Transcription-independent regulation of sting activation and innate immune responses by Irf8 in monocytes. Nat Commun. (2022) 13(1):4822. 10.1038/s41467-022-32401-1 35973990 PMC9381507

[6] Vaughan PS Aziz F van Wijnen AJ Wu S Harada H Taniguchi T Activation of a cell-cycle-regulated histone gene by the oncogenic transcription factor irf-2. Nature. (1995) 377(6547):362–5. 10.1038/377362a0 7566094

[7] Sun C Sun H Wei J Fan X Simon SI Passerini AG. Irf-1 regulates mitochondrial respiration and intrinsic apoptosis under metabolic stress through atp synthase ancillary factor Tmem70. Inflammation. (2024) 48(4):2548–62. 10.1007/s10753-024-02209-w 39641858

[8] Alfarano G Audano M Di Chiaro P Balestrieri C Milan M Polletti S Interferon regulatory factor 1 (Irf1) controls the metabolic programmes of low-grade pancreatic cancer cells. Gut. (2023) 72(1):109–28. 10.1136/gutjnl-2021-325811 35568393

[9] Lien C Fang CM Huso D Livak F Lu R Pitha PM. Critical role of irf-5 in regulation of B-cell differentiation. Proc Natl Acad Sci U S A. (2010) 107(10):4664–8. 10.1073/pnas.0911193107 20176957 PMC2842054

[10] Okon I Ding Y Zou MH. Ablation of interferon regulatory factor 3 promotes the stability of atherosclerotic plaques. Hypertension. (2017) 69(3):407–8. 10.1161/HYPERTENSIONAHA.116.08486 28115512 PMC5302192

[11] Fan X Li Q Wang Y Zhang DM Zhou J Chen Q Non-canonical nf-kappab contributes to endothelial pyroptosis and atherogenesis dependent on irf-1. Transl Res. (2023) 255(1878-1810):1–13. 10.1016/j.trsl.2022.11.001 36384204

[12] Gong X Liu Y Liu H Cao N Zeng L Tian M Re-analysis of single-cell transcriptomics reveals a critical role of macrophage-like smooth muscle cells in advanced atherosclerotic plaque. Theranostics. (2024) 14(4):1450–63. 10.7150/thno.87201 38389849 PMC10879858

[13] Thompson CD Matta B Barnes BJ. Therapeutic targeting of irfs: pathway-dependence or structure-based? Front Immunol. (2018) 9(1664-3224):2622. 10.3389/fimmu.2018.02622 30515152 PMC6255967

[14] Tamura T Yanai H Savitsky D Taniguchi T. The irf family transcription factors in immunity and oncogenesis. Annu Rev Immunol. (2008) 26(0732-0582):535–84. 10.1146/annurev.immunol.26.021607.090400 18303999

[15] Ning S Pagano JS Barber GN. Irf7: activation, regulation, modification and function. Genes Immun. (2011) 12(6):399–414. 10.1038/gene.2011.21 21490621 PMC4437765

[16] Qin BY Liu C Lam SS Srinath H Delston R Correia JJ Crystal structure of irf-3 reveals mechanism of autoinhibition and virus-induced phosphoactivation. Nat Struct Biol. (2003) 10(11):913–21. 10.1038/nsb1002 14555996

[17] Matta B Song S Li D Barnes BJ. Interferon regulatory factor signaling in autoimmune disease. Cytokine. (2017) 98(1096-0023):15–26. 10.1016/j.cyto.2017.02.006 28283223 PMC8033540

[18] Chen W Royer WE Jr. Structural insights into interferon regulatory factor activation. Cell Signal. (2010) 22(6):883–7. 10.1016/j.cellsig.2009.12.005 20043992 PMC2846214

[19] O'Neill LA Bowie AG. Sensing and signaling in antiviral innate immunity. Curr Biol. (2010) 20(7):R328–33. 10.1016/j.cub.2010.01.044 20392426

[20] Santana-de Anda K Gomez-Martin D Diaz-Zamudio M Alcocer-Varela J. Interferon regulatory factors: beyond the antiviral response and their link to the development of autoimmune pathology. Autoimmun Rev. (2011) 11(2):98–103. 10.1016/j.autrev.2011.08.006 21872684

[21] Taniguchi T Ogasawara K Takaoka A Tanaka N. Irf family of transcription factors as regulators of host defense. Annu Rev Immunol. (2001) 19(0732-0582):623–55. 10.1146/annurev.immunol.19.1.623 11244049

[22] Kong P Yang M Wang Y Yu KN Wu L Han W. Ferroptosis triggered by Stat1- Irf1-Acsl4 pathway was involved in radiation-induced intestinal injury. Redox Biol. (2023) 66(2213-2317):102857. 10.1016/j.redox.2023.102857 37611494 PMC10466894

[23] Rundberg Nilsson AJS Xian H Shalapour S Cammenga J Karin M. Irf1 regulates self-renewal and stress responsiveness to support hematopoietic stem cell maintenance. Sci Adv. (2023) 9(43):eadg5391. 10.1126/sciadv.adg5391 37889967 PMC10610924

[24] Li H Chen X Xu J Zhu L Li C Sun X Grp/grpr enhances alcohol-associated liver injury through the Irf1-mediated caspase-1 inflammasome and Nox2-dependent ros pathway. Hepatology. (2024) 79(2):392–408. 10.1097/HEP.0000000000000531 37409771

[25] Klune JR Dhupar R Kimura S Ueki S Cardinal J Nakao A Interferon regulatory factor-2 is protective against hepatic ischemia-reperfusion injury. Am J Physiol Gastrointest Liver Physiol. (2012) 303(5):G666–73. 10.1152/ajpgi.00050.2012 22744333 PMC3468551

[26] Lu J Bian ZY Zhang R Zhang Y Liu C Yan L Interferon regulatory factor 3 is a negative regulator of pathological cardiac hypertrophy. Basic Res Cardiol. (2013) 108(2):326. 10.1007/s00395-012-0326-9 23307144

[27] Wang J Li H Xue B Deng R Huang X Xu Y Irf1 promotes the innate immune response to viral infection by enhancing the activation of Irf3. J Virol. (2020) 94(22):e01231–20. 10.1128/JVI.01231-20 32878885 PMC7592201

[28] Huber M Lohoff M. Irf4 at the crossroads of effector T-cell fate decision. Eur J Immunol. (2014) 44(7):1886–95. 10.1002/eji.201344279 24782159

[29] Guo S Li ZZ Jiang DS Lu YY Liu Y Gao L Irf4 is a novel mediator for neuronal survival in ischaemic stroke. Cell Death Differ. (2014) 21(6):888–903. 10.1038/cdd.2014.9 24510125 PMC4013523

[30] Gupta S Jiang M Anthony A Pernis AB. Lineage-Specific modulation of interleukin 4 signaling by interferon regulatory factor 4. J Exp Med. (1999) 190(12):1837–48. 10.1084/jem.190.12.1837 10601358 PMC2195723

[31] Eguchi J Kong X Tenta M Wang X Kang S Rosen ED. Interferon regulatory factor 4 regulates obesity-induced inflammation through regulation of adipose tissue macrophage polarization. Diabetes. (2013) 62(10):3394–403. 10.2337/db12-1327 23835343 PMC3781469

[32] Wong RWJ Ong JZL Theardy MS Sanda T. Irf4 as an oncogenic master transcription factor. Cancers (Basel). (2022) 14(17):4314. 10.3390/cancers14174314 36077849 PMC9454692

[33] Xu WD Pan HF Xu Y Ye DQ. Interferon regulatory factor 5 and autoimmune lupus. Expert Rev Mol Med. (2013) 15(1462-3994):e6. 10.1017/erm.2013.7 23883595

[34] Saliba DG Heger A Eames HL Oikonomopoulos S Teixeira A Blazek K Irf5:rela interaction targets inflammatory genes in macrophages. Cell Rep. (2014) 8(5):1308–17. 10.1016/j.celrep.2014.07.034 25159141 PMC4471814

[35] Krausgruber T Blazek K Smallie T Alzabin S Lockstone H Sahgal N Irf5 promotes inflammatory macrophage polarization and Th1-Th17 responses. Nat Immunol. (2011) 12(3):231–8. 10.1038/ni.1990 21240265

[36] Takaoka A Yanai H Kondo S Duncan G Negishi H Mizutani T Integral role of irf-5 in the gene induction programme activated by toll-like receptors. Nature. (2005) 434(7030):243–9. 10.1038/nature03308 15665823

[37] Garcia-Bermudez M Lopez-Mejias R Genre F Castaneda S Llorca J Gonzalez-Juanatey C Interferon regulatory factor 5 genetic variants are associated with cardiovascular disease in patients with rheumatoid arthritis. Arthritis Res Ther. (2014) 16(4):R146. 10.1186/ar4608 25011482 PMC4227041

[38] Agca R van Sijl AM Vosslamber S Voskuyl AE Verweij CL Nurmohamed MT. Interferon regulatory factor 5 gene variants Rs2004640 and Rs4728142 are associated with carotid intima media thickness but not with cardiovascular events in rheumatoid arthritis. Clin Exp Rheumatol. (2022) 40(1):64–8. 10.55563/clinexprheumatol/pf511x 33666161

[39] Chung CP Solus JF Oeser A Li C Raggi P Smith JR Genetic variation and coronary atherosclerosis in patients with systemic lupus erythematosus. Lupus. (2014) 23(9):876–80. 10.1177/0961203314530019 24699314 PMC4185017

[40] Posadas-Sanchez R Cardoso-Saldana G Fragoso JM Vargas-Alarcon G. Interferon regulatory factor 5 (Irf5) gene haplotypes are associated with premature coronary artery disease. Association of the Irf5 polymorphisms with cardiometabolic parameters. The genetics of atherosclerotic disease (gea) Mexican study. Biomolecules. (2021) 11(3):443. 10.3390/biom11030443 33802675 PMC8002496

[41] Oberbeck N Pham VC Webster JD Reja R Huang CS Zhang Y The Ripk4-Irf6 signalling axis safeguards epidermal differentiation and barrier function. Nature. (2019) 574(7777):249–53. 10.1038/s41586-019-1615-3 31578523

[42] Kim IK Diamond MS Yuan S Kemp SB Kahn BM Li Q Plasticity-induced repression of Irf6 underlies acquired resistance to cancer immunotherapy in pancreatic ductal adenocarcinoma. Nat Commun. (2024) 15(1):1532. 10.1038/s41467-024-46048-7 38378697 PMC10879147

[43] Lu J Liu X Zheng J Song J Liu Y Ruan X Lin28a promotes Irf6-regulated aerobic glycolysis in glioma cells by stabilizing Snhg14. Cell Death Dis. (2020) 11(6):447. 10.1038/s41419-020-2650-6 32527996 PMC7289837

[44] Ma W Huang G Wang Z Wang L Gao Q. Irf7: role and regulation in immunity and autoimmunity. Front Immunol. (2023) 14(1664-3224):1236923. 10.3389/fimmu.2023.1236923 37638030 PMC10449649

[45] Mummidi S Kuroda M Nishiguchi M Ugawa N Ishikawa E Kawabata Y Interferon regulatory factor 7 mediates obesity-associated mcp-1 transcription. PLoS One. (2020) 15(5):e0233390. 10.1371/journal.pone.0233390 32437400 PMC7241760

[46] Nezos A Evangelopoulos ME Mavragani CP. Genetic contributors and soluble mediators in prediction of autoimmune comorbidity. J Autoimmun. (2019) 104(1095-9157):102317. 10.1016/j.jaut.2019.102317 31444033

[47] Leonard D Svenungsson E Sandling JK Berggren O Jonsen A Bengtsson C Coronary heart disease in systemic lupus erythematosus is associated with interferon regulatory factor-8 gene variants. Circ Cardiovasc Genet. (2013) 6(3):255–63. 10.1161/CIRCGENETICS.113.000044 23661672

[48] Crosslin DR McDavid A Weston N Zheng X Hart E de Andrade M Genetic variation associated with circulating monocyte count in the emerge network. Hum Mol Genet. (2013) 22(10):2119–27. 10.1093/hmg/ddt010 23314186 PMC3633369

[49] Zhang Q Kisand K Feng Y Rinchai D Jouanguy E Cobat A In search of a function for human type iii interferons: insights from inherited and acquired deficits. Curr Opin Immunol. (2024) 87(1879-0372):102427. 10.1016/j.coi.2024.102427 38781720 PMC11209856

[50] Wang XA Zhang R Jiang D Deng W Zhang S Deng S Interferon regulatory factor 9 protects against hepatic insulin resistance and steatosis in male mice. Hepatology. (2013) 58(2):603–16. 10.1002/hep.26368 23471885 PMC3736732

[51] Huang R Hu Z Chen X Cao Y Li H Zhang H The transcription factor Sub1 is a master regulator of the macrophage tlr response in atherosclerosis. Adv Sci (Weinh). (2021) 8(19):e2004162. 10.1002/advs.202004162 34378353 PMC8498911

[52] Liu H Cheng WL Jiang X Wang PX Fang C Zhu XY Ablation of interferon regulatory factor 3 protects against atherosclerosis in apolipoprotein E-deficient mice. Hypertension. (2017) 69(3):510–20. 10.1161/HYPERTENSIONAHA.116.08395 28115514

[53] Leipner J Dederichs TS von Ehr A Rauterberg S Ehlert C Merz J Myeloid cell-specific Irf5 deficiency stabilizes atherosclerotic plaques in apoe(-/-) mice. Mol Metab. (2021) 53(2212-8778):101250. 10.1016/j.molmet.2021.101250 33991749 PMC8178123

[54] Lv JJ Wang H Zhang C Zhang TJ Wei HL Liu ZK Cd147 sparks atherosclerosis by driving M1 phenotype and impairing efferocytosis. Circ Res. (2024) 134(2):165–85. 10.1161/CIRCRESAHA.123.323223 38166463

[55] Wu Z Geng J Bai Y Qi Y Chang C Jiao Y Microrna-22 inhibition promotes the development of atherosclerosis via targeting interferon regulator factor 5. Exp Cell Res. (2021) 409(2):112922. 10.1016/j.yexcr.2021.112922 34780785

[56] Senatus L Lopez-Diez R Egana-Gorrono L Liu J Hu J Daffu G Rage impairs murine diabetic atherosclerosis regression and implicates Irf7 in macrophage inflammation and cholesterol metabolism. JCI Insight. (2020) 5(13):e137289. 10.1172/jci.insight.137289 32641587 PMC7406264

[57] Doring Y Soehnlein O Drechsler M Shagdarsuren E Chaudhari SM Meiler S Hematopoietic interferon regulatory factor 8-deficiency accelerates atherosclerosis in mice. Arterioscler Thromb Vasc Biol. (2012) 32(7):1613–23. 10.1161/ATVBAHA.111.236539 22556330

[58] Gimbrone MA Jr Garcia-Cardena G. Endothelial cell dysfunction and the pathobiology of atherosclerosis. Circ Res. (2016) 118(4):620–36. 10.1161/CIRCRESAHA.115.306301 26892962 PMC4762052

[59] Dagia NM Harii N Meli AE Sun X Lewis CJ Kohn LD Phenyl methimazole inhibits tnf-alpha-induced vcam-1 expression in an ifn regulatory factor-1-dependent manner and reduces monocytic cell adhesion to endothelial cells. J Immunol. (2004) 173(3):2041–9. 10.4049/jimmunol.173.3.2041 15265939

[60] Neish AS Read MA Thanos D Pine R Maniatis T Collins T. Endothelial interferon regulatory factor 1 cooperates with nf-kappa B as a transcriptional activator of vascular cell adhesion molecule 1. Mol Cell Biol. (1995) 15(5):2558–69. 10.1128/MCB.15.5.2558 7537851 PMC230486

[61] Sun C Alkhoury K Wang YI Foster GA Radecke CE Tam K Irf-1 and Mirna126 modulate vcam-1 expression in response to a high-fat meal. Circ Res. (2012) 111(8):1054–64. 10.1161/CIRCRESAHA.112.270314 22874466 PMC3810165

[62] Wang YI Bettaieb A Sun C DeVerse JS Radecke CE Mathew S Triglyceride-rich lipoprotein modulates endothelial vascular cell adhesion molecule (vcam)-1 expression via differential regulation of endoplasmic reticulum stress. PLoS One. (2013) 8(10):e78322. 10.1371/journal.pone.0078322 24205197 PMC3804477

[63] Tamargo IA Baek KI Kim Y Park C Jo H. Flow-induced reprogramming of endothelial cells in atherosclerosis. Nat Rev Cardiol. (2023) 20(11):738–53. 10.1038/s41569-023-00883-1 37225873 PMC10206587

[64] DeVerse JS Sandhu AS Mendoza N Edwards CM Sun C Simon SI Shear stress modulates vcam-1 expression in response to tnf-alpha and dietary lipids via interferon regulatory factor-1 in cultured endothelium. Am J Physiol Heart Circ Physiol. (2013) 305(8):H1149–57. 10.1152/ajpheart.00311.2013 23934855 PMC3798784

[65] Bailey KA Moreno E Haj FG Simon SI Passerini AG. Mechanoregulation of P38 activity enhances endoplasmic Reticulum stress-mediated inflammation by arterial endothelium. FASEB J. (2019) 33(11):12888–99. 10.1096/fj.201900236R 31499005 PMC6902662

[66] Qian Z Shaofang F Chen C Chunhua S Nan W Chao L. Il-33 suppresses the progression of atherosclerosis via the Erk1/2-Irf1-vcam-1 pathway. Cardiovasc Drugs Ther. (2024) 38(3):569–80. 10.1007/s10557-023-07523-3 37957490

[67] Li Y Zhang Q Li N Ding L Yi J Xiao Y Ataxin-10 inhibits tnf-alpha-induced endothelial inflammation via suppressing interferon regulatory factor-1. Mediators Inflamm. (2021) 2021(1466-1861):7042148. 10.1155/2021/7042148 34858081 PMC8632433

[68] Wagner AH Gebauer M Pollok-Kopp B Hecker M. Cytokine-inducible Cd40 expression in human endothelial cells is mediated by interferon regulatory factor-1. Blood. (2002) 99(2):520–5. 10.1182/blood.v99.2.520 11781233

[69] Wang F Xia W Liu F Li J Wang G Gu J. Interferon regulator factor 1/retinoic inducible gene I (Irf1/rig-I) axis mediates 25-hydroxycholesterol-induced interleukin-8 production in atherosclerosis. Cardiovasc Res. (2012) 93(1):190–9. 10.1093/cvr/cvr260 21979142

[70] Guo M Mao X Ji Q Lang M Li S Peng Y Inhibition of ifn regulatory factor-1 down-regulate Th1 cell function in patients with acute coronary syndrome. J Clin Immunol. (2010) 30(2):241–52. 10.1007/s10875-010-9367-8 20177960

[71] Guo M Yan R Wang C Shi H Sun M Guo S Ifn regulatory factor-1 modulates the function of dendritic cells in patients with acute coronary syndrome. Cell Physiol Biochem. (2015) 36(2):599–610. 10.1159/000430123 25997853

[72] Li X Chen X Zheng L Chen M Zhang Y Zhu R Non-canonical sting-perk pathway dependent epigenetic regulation of vascular endothelial dysfunction via integrating Irf3 and nf-kappab in inflammatory response. Acta Pharm Sin B. (2023) 13(12):4765–84. 10.1016/j.apsb.2023.08.015 38045042 PMC10692388

[73] Sakai C Ueda K Goda K Fujita R Maeda J Nakayama S A possible role for proinflammatory activation via cgas-sting pathway in atherosclerosis induced by accumulation of DNA double-strand breaks. Sci Rep. (2023) 13(1):16470. 10.1038/s41598-023-43848-7 37777633 PMC10542807

[74] Gravel S-P Servant MJ. Roles of an i*κ*b kinase-related pathway in human cytomegalovirus-infected vascular smooth muscle cells. J Biol Chem. (2005) 280(9):7477–86. 10.1074/jbc.M410392200 15619605

[75] Liu N Liu JT Ji YY Lu PP. C-reactive protein triggers inflammatory responses partly via Tlr4/Irf3/nf-kappab signaling pathway in rat vascular smooth muscle cells. Life Sci. (2010) 87(11-12):367–74. 10.1016/j.lfs.2010.07.012 20670634

[76] Ji Y Liu J Wang Z Li Z. Ppargamma agonist rosiglitazone ameliorates lps-induced inflammation in vascular smooth muscle cells via the Tlr4/trif/Irf3/ip-10 signaling pathway. Cytokine. (2011) 55(3):409–19. 10.1016/j.cyto.2011.05.020 21700474

[77] Xing Z Zhen Y Chen J Du M Li D Liu R Kpna2 silencing, regulated by E3 ubiquitin ligase Fbxw7, alleviates endothelial dysfunction and inflammation through inhibiting the nuclear translocation of P65 and Irf3: a possible therapeutic approach for atherosclerosis. Inflammation. (2023) 46(6):2071–88. 10.1007/s10753-023-01863-w 37432596

[78] Seneviratne AN Edsfeldt A Cole JE Kassiteridi C Swart M Park I Interferon regulatory factor 5 controls necrotic core formation in atherosclerotic lesions by impairing efferocytosis. Circulation. (2017) 136(12):1140–54. 10.1161/CIRCULATIONAHA.117.027844 28698173 PMC5598917

[79] Edsfeldt A Swart M Singh P Dib L Sun J Cole JE Interferon regulatory factor-5-dependent Cd11c+ macrophages contribute to the formation of rupture-prone atherosclerotic plaques. Eur Heart J. (2022) 43(19):1864–77. 10.1093/eurheartj/ehab920 35567557 PMC9113304

[80] Watkins AA Yasuda K Wilson GE Aprahamian T Xie Y Maganto-Garcia E Irf5 deficiency ameliorates lupus but promotes atherosclerosis and metabolic dysfunction in a mouse model of lupus-associated atherosclerosis. J Immunol. (2015) 194(4):1467–79. 10.4049/jimmunol.1402807 25595782 PMC4323680

[81] Pourcet B Feig JE Vengrenyuk Y Hobbs AJ Kepka-Lenhart D Garabedian MJ Lxralpha regulates macrophage arginase 1 through pu.1 and interferon regulatory factor 8. Circ Res. (2011) 109(5):492–501. 10.1161/CIRCRESAHA.111.241810 21757649 PMC3180895

[82] Clement M Haddad Y Raffort J Lareyre F Newland SA Master L Deletion of Irf8 (interferon regulatory factor 8)-dependent dendritic cells abrogates proatherogenic adaptive immunity. Circ Res. (2018) 122(6):813–20. 10.1161/CIRCRESAHA.118.312713 29436389

[83] Yan J Horng T. Lipid metabolism in regulation of macrophage functions. Trends Cell Biol. (2020) 30(12):979–89. 10.1016/j.tcb.2020.09.006 33036870

[84] Holvoet P Davey PC De Keyzer D Doukoure M Deridder E Bochaton-Piallat ML Oxidized low-density lipoprotein correlates positively with toll-like receptor 2 and interferon regulatory factor-1 and inversely with superoxide dismutase-1 expression: studies in hypercholesterolemic swine and thp-1 cells. Arterioscler Thromb Vasc Biol. (2006) 26(7):1558–65. 10.1161/01.ATV.0000226553.01555.02 16690872

[85] Zhu Y Xu Y Han D Zhang X Qin C Liu J Scavenger receptor-ai targeted theranostic nanoparticles for regression of atherosclerotic plaques via Abca1 modulation. Nanomedicine. (2023) 50(1549-9642):102672. 10.1016/j.nano.2023.102672 37044196

[86] Wang J Xiao Q Wang L Wang Y Wang D Ding H. Role of Abca1 in cardiovascular disease. J Pers Med. (2022) 12(6):1010. 10.3390/jpm12061010 35743794 PMC9225161

[87] Castrillo A Joseph SB Vaidya SA Haberland M Fogelman AM Cheng G Crosstalk between lxr and toll-like receptor signaling mediates bacterial and viral antagonism of cholesterol metabolism. Mol Cell. (2003) 12(4):805–16. 10.1016/s1097-2765(03)00384-8 14580333

[88] Chen S Sorrentino R Shimada K Bulut Y Doherty TM Crother TR Chlamydia pneumoniae-induced foam cell formation requires Myd88-dependent and -independent signaling and is reciprocally modulated by liver X receptor activation. J Immunol. (2008) 181(10):7186–93. 10.4049/jimmunol.181.10.7186 18981140 PMC2662697

[89] Shu H Peng Y Hang W Nie J Zhou N Wang DW. The role of Cd36 in cardiovascular disease. Cardiovasc Res. (2022) 118(1):115–29. 10.1093/cvr/cvaa319 33210138 PMC8752351

[90] Sorrentino R Morello S Chen S Bonavita E Pinto A. The activation of liver X receptors inhibits toll-like receptor-9-induced foam cell formation. J Cell Physiol. (2010) 223(1):158–67. 10.1002/jcp.22022 20049870

[91] Wei Y Lan B Zheng T Yang L Zhang X Cheng L Gsdme-mediated pyroptosis promotes the progression and associated inflammation of atherosclerosis. Nat Commun. (2023) 14(1):929. 10.1038/s41467-023-36614-w 36807553 PMC9938904

[92] Guo M Yan R Yao H Duan L Sun M Xue Z Ifn regulatory factor 1 mediates macrophage pyroptosis induced by oxidized low-density lipoprotein in patients with acute coronary syndrome. Mediators Inflamm. (2019) 2019(1466-1861):2917128. 10.1155/2019/2917128 31871426 PMC6913184

[93] Franchi L Eigenbrod T Munoz-Planillo R Nunez G. The inflammasome: a caspase-1-activation platform that regulates immune responses and disease pathogenesis. Nat Immunol. (2009) 10(3):241–7. 10.1038/ni.1703 19221555 PMC2820724

[94] Guo M Yan R Ji Q Yao H Sun M Duan L Ifn regulatory factor-1 induced macrophage pyroptosis by modulating M6a modification of circ_0029589 in patients with acute coronary syndrome. Int Immunopharmacol. (2020) 86(1529-2916):106800. 10.1016/j.intimp.2020.106800 32674051

[95] Grootaert MOJ Bennett MR. Vascular smooth muscle cells in atherosclerosis: time for a re-assessment. Cardiovasc Res. (2021) 117(11):2326–39. 10.1093/cvr/cvab046 33576407 PMC8479803

[96] Zhang F Guo X Xia Y Mao L. An update on the phenotypic switching of vascular smooth muscle cells in the pathogenesis of atherosclerosis. Cell Mol Life Sci. (2021) 79(1):6. 10.1007/s00018-021-04079-z 34936041 PMC11072026

[97] Tanner FC Meier P Greutert H Champion C Nabel EG Luscher TF. Nitric oxide modulates expression of cell cycle regulatory proteins: a cytostatic strategy for inhibition of human vascular smooth muscle cell proliferation. Circulation. (2000) 101(16):1982–9. 10.1161/01.cir.101.16.1982 10779466

[98] Quyyumi AA Dakak N Andrews NP Gilligan DM Panza JA Cannon RO 3rd. Contribution of nitric oxide to metabolic coronary vasodilation in the human heart. Circulation. (1995) 92(3):320–6. 10.1161/01.cir.92.3.320 7634444

[99] Poon M Marx SO Gallo R Badimon JJ Taubman MB Marks AR. Rapamycin inhibits vascular smooth muscle cell migration. J Clin Invest. (1996) 98(10):2277–83. 10.1172/JCI119038 8941644 PMC507677

[100] Wessely R Hengst L Jaschke B Wegener F Richter T Lupetti R A central role of interferon regulatory factor-1 for the limitation of neointimal hyperplasia. Hum Mol Genet. (2003) 12(2):177–87. 10.1093/hmg/ddg018 12499398

[101] Shen Y Sun Z Mao S Zhang Y Jiang W Wang H. Irf-1 contributes to the pathological phenotype of vsmcs during atherogenesis by increasing Ccl19 transcription. Aging (Albany NY. (2020) 13(1):933–43. 10.18632/aging.202204 33424012 PMC7835033

[102] Mittal B Mishra A Srivastava A Kumar S Garg N. Matrix metalloproteinases in coronary artery disease. Adv Clin Chem. (2014) 64(0065-2423):1–72. 10.1016/b978-0-12-800263-6.00001-x 24938016

[103] Newby AC. Metalloproteinases and vulnerable atherosclerotic plaques. Trends Cardiovasc Med. (2007) 17(8):253–8. 10.1016/j.tcm.2007.09.001 18021934 PMC2850988

[104] Pugliese G Iacobini C Fantauzzi CB Menini S. The dark and bright Side of atherosclerotic calcification. Atherosclerosis. (2015) 238(2):220–30. 10.1016/j.atherosclerosis.2014.12.011 25528431

[105] Yap C Mieremet A de Vries CJM Micha D de Waard V. Six shades of vascular smooth muscle cells illuminated by Klf4 (krüppel-like factor 4). Arterioscler Thromb Vasc Biol. (2021) 41(11):2693–707. 10.1161/atvbaha.121.316600 34470477 PMC8545254

